# Relationship between systolic blood pressure and renal function on clinical outcomes in patients with atrial fibrillation: a report from the prospective AF-GEN-UK Registry

**DOI:** 10.1097/HJH.0000000000003856

**Published:** 2024-09-23

**Authors:** Alena Shantsila, Gregory Y.H. Lip, Deirdre A. Lane

**Affiliations:** a Department of Cardiovascular and Metabolic Medicine, Institute of Life Course and Medical Sciences, University of Liverpool; b Liverpool Centre for Cardiovascular Science, University of Liverpool, Liverpool John Moores University and Liverpool Heart and Chest Hospital, Liverpool, UK; c Department of Clinical Medicine, Aalborg University, Aalborg, Denmark

**Keywords:** atrial fibrillation, blood pressure, mortality, prospective, renal function

## Abstract

**Background:**

Blood pressure (BP) extremes and renal (dys)function contribute to poor outcomes in patients with atrial fibrillation (AF). Using data from the prospective AF-GEN-UK study, we investigated the effect of systolic BP and interaction with renal function for prognostication.

**Methods:**

Baseline systolic BP (SBP) values were recorded for 1580 patients (mean [SD] age 71 [11] years, 60% male) and categorized as follows: 120–129 mmHg (*n* = 289, reference group) <110 mmHg (*n* = 165), 110–119 mmHg, (*n* = 254), 130–139 mmHg (*n* = 321), 140–159 mmHg (*n* = 385) and ≥160 mmHg (*n* = 166). Cox regression analysis, adjusted for age, oral anticoagulation (OAC) and CHA_2_DS_2_-VASc score established the impact of SBP, renal function and their interaction on 1-year outcomes. SBP groups were compared using ANOVA and chi-square tests.

**Results:**

OAC use was 84% and similar across SBP groups. Renal dysfunction [estimated baseline glomerular filtration rate (eGFR) < 60 ml/min] was present in 24%, with significantly lower eGFR values in the SBP 110–119 mmHg group. History of heart failure was significantly higher in those with SBP <110 mmHg. SBP <110 mmHg was predictive of all cause-death on univariate [hazard ratio (HR) 2.36, 95% confidence interval (CI) 1.20–4.64] and adjusted (aHR 9.71, 95% CI 1.73–54.5) regression. There was no statistically significant interaction between SBP and eGFR, no associations of SBP with haemorrhagic or thromboembolic events.

**Conclusions:**

In people with AF, SBP <110 mmHg was independently predictive of all-cause death, with no significant interaction between SBP and renal (dys)function. This may reflect general poor health and/or excessive antihypertensive therapy, which should be avoided.

## INTRODUCTION

Elevated systolic blood pressure (BP) is the most significant modifiable risk factor for increased morbidity and mortality [1]. From 1990 to 2019, the number of disability-adjusted life years due to elevated systolic BP increased from 154 million to 235 million, while the number of premature deaths rose to 10.8 million [2]. There are many pathways linking hypertension to poor health outcomes and optimal BP levels may be influenced by co-morbidities, although these interactions remain unclear. Elevated BP contributes significantly to incident atrial fibrillation (AF) [3,4]. The risk of AF among patients with hypertension is 1.7-fold higher than in normotensive individuals [5]. When AF occurs, patients with hypertension are more prone to developing debilitating complications, such as stroke, heart failure and advanced chronic kidney disease [6,7]. The European Society of Cardiology (ESC) guidelines on AF management recommend a target systolic BP of 120–129 mmHg as this level is associated with the lowest risk of poor outcomes [8,9].

The incidence of both hypertension and AF increases with age, contributing to the healthcare burden, and evidence shows that systolic BP, rather than diastolic BP, is a better predictor of outcomes in patients >50 years old [10–12]. Both hypertension and AF negatively affect renal function, another important health outcome prognosticator. However, the interaction between systolic BP and renal function in patients with AF remains unclear, and the optimal systolic BP in AF remains uncertain from this aspect. Hence, the necessity of active BP monitoring and early identification of deterioration of kidney function, as well as a better understanding of how the level of renal function influences the risk of AF complications in people with different BP levels in real-world settings.

The UK extension of the EURObservational Research Programme (EORP) Long-term General Registry of patients with AF, the AF-GEN-UK study, assessed the contemporary management of AF in primary and secondary care in the UK [13]. In this analysis, we assessed the combined impact of systolic BP levels and renal (dys)function and their possible interaction on mortality, thromboembolic and haemorrhagic events in patients with AF from the AF-GEN-UK study.

## METHODS

The AF-GEN-UK is a prospective multicentre registry in the UK, that enrolled patients with AF from 101 primary and secondary care sites [13], collecting data on the real-world management of AF by cardiologists, general practitioners, stroke physicians, acute medicine and emergency medicine physicians at the time of enrolment and at 12-month follow-up. The study was approved by the West Midlands Research Ethics Committee (17/WM/0013) and all patients provided written informed consent.

Participants were consecutively recruited during the 12-month period from June 2017 to June 2018 from 43 hospitals and 58 general practices in the UK. Eligible adult patients had a qualifying episode of AF documented by electrocardiogram (ECG) within the previous 12 months. Patients with atrial flutter but without AF were excluded. Clinical assessment was performed at baseline and further health data was collected using an electronic case report form (eCRF). Additionally, quality of life was assessed using the EQ-5D questionnaire [14] at baseline and during the 12-month follow-up.

The outcomes of interest were all-cause death, major bleeding events (intracranial and major extracranial bleeding) and thromboembolic events [ischaemic stroke, transient ischaemic attack (TIA), pulmonary embolism, deep vein thrombosis (DVT)] during the first 12-months from baseline.

Systolic and diastolic blood pressure (BP) values were collected as part of routine clinical assessment. Clinicians were expected to adhere to the official UK NICE recommendations on BP measurement [15]. For this analysis, patients were grouped according to their baseline systolic BP: 120–129 mmHg (reference group), <110 mmHg, 110–119 mmHg, 130–139 mmHg, 140–159 mmHg and ≥160 mmHg.

### Statistical analysis

The distribution of the continuous data was assessed using Kolmogorov-Smirnov test and histogram visualization. Variables were compared between the systolic BP groups. Missing values were labelled as missing with no additional statistical tests performed to account for missing data. Normally distributed continuous variables were compared using one-way ANOVA and presented as mean and standard deviation (SD). Nonnormally distributed variables were compared using Kruskal–Wallis test and reported as median and interquartile range. Categorical variables were compared using the chi-squared test and expressed as absolute numbers and frequency (percentage of the group). Prevalence of the outcomes of interest were reported as a percentage and absolute number.

Kaplan–Meier curves were constructed for all-cause death by systolic BP groups. Multivariate Cox regression analysis was conducted to calculate unadjusted hazard ratios (HR), adjusted hazard ratios (aHR) and 95% confidence intervals (CIs) for the study outcomes. Model 1, exploring the impact of systolic BP (using systolic BP groups) was adjusted for age, use of oral anticoagulation (OAC), CHA_2_DS_2_-VASc score, estimated baseline glomerular filtration rate (eGFR) and their interaction. Model 2, exploring the impact of diastolic BP (using diastolic BP categories: 70–79 mmHg (reference), <60 mmHg, 60–69 mmHg, 80–89 mmHg, and ≥90 mmHg) was adjusted for age, use of OAC, CHA_2_DS_2_-VASc score, eGFR and their interaction. A two-tailed *P*-value <0.05 was considered statistically significant. Analyses were performed using STATA statistical software, version 13 (STATA Inc., USA).

## RESULTS

Among 1595 participants of the AF-GEN-UK registry, 1580 (99.1%) patients had systolic BP available at baseline and were included in the current analysis. The overall mean (SD) age was 70.6 (11.2) years, 950 (60.1%) males, and most (1538, 97.3%) were of white origin.

The baseline clinical and demographic characteristics by systolic BP group are shown in Table 1. Patients with uncontrolled systolic BP (>140 mmHg) were older compared to the reference group (all *P* < 0.01), had higher diastolic and mean arterial pressure, included more women and a higher proportion of patients with diagnosed hypertension, and had correspondingly higher CHA_2_DS_2_VASc scores. There were no significant differences in ethnicity, alcohol use and smoking status between the study groups. Prevalence of heart failure was highest (37.9%) in people with systolic BP <110 mmHg. The prevalence of diabetes mellitus, coronary artery disease, peripheral vascular disease, and previous thromboembolic events was similar between groups. Overall, renal function, assessed by eGFR, was preserved across the systolic BP groups, with lower eGFR values of 73.1 ml/min observed in the group with systolic BP 110–119 mmHg; this group had the highest proportion (6.5%) of patients with eGFR <30 ml/min. Newly diagnosed AF was the most common type of AF (43.3%), with significantly higher rates among those with systolic BP ≥160 mmHg (50.5%) and <110 mmHg (51.5%). There were no significant differences in the rates of symptomatic AF across study groups.

**TABLE 1:** Demographic and clinical characteristics of participants at time of enrolment by systolic blood pressure group

	Study groups according to baseline systolic blood pressure							
Variables Mean (SD), *n* (%)	All groups (*n* = 1580)	120–129 mmHg^a^ (*n* = 289)	<110 mmHg (*n* = 165)	110–119 mmHg (*n* = 254)	130–139 mmHg (*n* = 321)	140–159 mmHg (*n* = 385)	≥160 mmHg (*n* = 166)	*P*-value
Age, years	70.6 (11.2)	68.5 (12.5)	70.5 (11.4)	70.8 (11.7)	69.8 (11.0)	71.6 (10.1)	72.7 (10.2)	**<0.001**
<65	407 (25.8)	94 (32.5)	48 (29.1)	68 (26.8)	90 (28.0)	77 (20.0)	30 (18.1)	**<0.001**
65-74	567 (35.9)	109 (37.7)	52 (31.5)	84 (33.1)	111 (34.6)	157 (40.8)	54 (32.5)	
≥75	606 (38.4)	85 (29.8)	65 (39.4)	102 (40.2)	120 (37.4)	151 (39.2)	82 (49.4)	
Male	950 (60.1)	181 (62.6)	104 (63.0)	152 (59.8)	217 (67.6)	214 (55.6)	82 (49.4)	**0.001**
Ethnicity								0.55
White	1538 (97.3)	282 (97.6)	160 (97.0)	248 (97.6)	312 (97.2)	376 (97.7)	160 (96.4)	
Black	9 (0.6)	4 (1.4)	0	0	2 (0.6)	1 (0.3)	2 (1.2)	
South Asian	12 (0.8)	3 (1.0)	2 (1.2)	2 (0.8)	2 (0.6)	1 (0.3)	2 (1.2)	
Other	6 (0.4)	0	0	2 (0.8)	1 (0.3)	2 (0.5)	1 (0.6)	
Unknown	15 (0.9)	0	3 (1.8)	2 (0.8)	4 (1.3)	5 (1.3)	1 (0.6)	
Never smoked (*n* = 1579)	774 (49.0)	144 (50.0)	80 (48.5)	120 (47.2)	159 (49.5)	192 (49.9)	79 (47.0)	1.0
eGFR, ml/min (*n* = 1402), median [interquartile range]	80.5[60.9- 107.4]	84.0 [62.9- 108.8]	77.7 [60.1- 109.0]	73.1 [55.8- 102.2]	89.3 [63.2- 114.0]	79.1 [61.2- 106.4]	76.9 [58.9- 99.9]	**0.001**
Mean arterial pressure	96.9 (14.3)	92.1 (7.6)	77.5 (7.7)	86.2 (7.4)	98.3 (7.6)	105.2 (8.7)	119.3 (12.5)	**<0.001**
Diastolic blood pressure	78.9 (13.8)	76.2 (11.0)	65.3 (9.9)	72.1 (10.5)	80.5 (11.2)	84.4 (12.3)	91.7 (15.8)	**<0.001**
CKD groups (*n* = 1402)								**0.001**
≥90, ml/min	580 (41.4)	116 (44.4)	55 (36.0)	79 (36.6)	140 (49.3)	133 (39.7)	57 (37.3)	
60–89 ml/min	489 (34.9)	89 (34.1)	60 (39.2)	70 (32.4)	88 (31.0)	126 (37.6)	56 (36.6)	
45–59 ml/min	194 (13.8)	35 (13.4)	20 (13.1)	33 (15.3)	39 (13.7)	46 (13.7)	21 (13.7)	
30–44 ml/min	107 (7.6)	18 (6.9)	14 (9.2)	20 (9.3)	13 (4.6)	29 (8.7)	13 (8.5)	
<30 ml/min	32 (2.3)	3 (1.2)	4 (2.6)	14 (6.5)	4 (1.4)	1 (0.3)	6 (3.9)	
Past medical history								
Hypertension (*n* = 1021)	733 (71.8)	123 (62.8)	52 (63.4)	102 (68.9)	154 (72.0)	199 (78.0)	103 (81.8)	**<0.001**
Diabetes (*n* = 1572)	301 (19.2)	63 (21.9)	32 (19.6)	50 (19.8)	61 (19.0)	67 (17.5)	28 (17.0)	0.76
Heart failure (*n* = 1530)	292 (19.1)	51 (18.5)	61 (37.9)	64 (26.3)	51 (16.1)	50 (1.3)	15 (9.5)	**<0.001**
Coronary artery disease (*n* = 1494)	306 (20.5)	48 (17.7)	38 (25.0)	57 (23.9)	51 (16.6)	74 (20.3)	38 (23.8)	0.12
Peripheral vascular disease (*n* = 1558)	50 (3.2)	5 (1.8)	7 (4.3)	6 (2.5)	9 (2.8)	15 (3.9)	8 (4.9)	0.39
Previous thrombo-embolism (*n* = 1550)	263 (17.0)	46 (16.5)	18 (11.0)	37 (14.9)	50 (15.8)	79 (20.7)	33 (20.4)	0.07
CHA_2_DS_2_-VASc score (*n* = 1577)	2.8 (1.7)	2.6 (1.8)	2.8 (1.3)	2.8 (1.7)	2.7 (1.6)	3.0 (1.7)	3.3 (1.6)	**0.003**
HAS-BLED score (*n* = 1593)	1.5 (1.0)	1.3 (1.0)	1.3 (0.9)	1.4 (1.0)	1.4 (0.9)	1.6 (0.9)	2.4 (0.9)	**<0.001**
Type of AF (*n* = 1568)								**<0.001**
First diagnosed	679 (43.3)	119 (41.6)	85 (51.5)	108 (42.7)	120 (37.6)	164 (43.0)	83 (50.6)	
Paroxysmal	269 (17.2)	59 (20.6)	19 (11.5)	32 (12.7)	51 (16.0)	65 (17.1)	43 (26.2)	
Persistent	416 (26.5)	76 (26.6)	37 (22.4)	82 (32.4)	90 (28.2)	102 (26.8)	29 (17.7)	
Permanent	204 (13.0)	32 (11.2)	24 (14.6)	31 (12.3)	58 (18.2)	50 (13.1)	9 (5.5)	
EHRA score (*n* = 1578)								0.08
EHRA I	702 (44.5)	120 (41.7)	64 (38.8)	113 (44.5)	152 (47.4)	178 (46.4)	75 (45.2)	
EHRA II	590 (37.4)	107 (37.2)	64 (38.8)	80 (31.5)	128 (39.9)	147 (38.3)	64 (38.6)	
EHRA III	257 (16.3)	55 (19.1)	31 (18.8)	56 (22.1)	37 (11.5)	53 (13.8)	25 (15.1)	
EHRA IV	29 (1.8)	6 (2.1)	6 (3.6)	5 (2.0)	4 (1.3)	6 (1.6)	2 (1.2)	

AF, atrial fibrillation; CHA_2_DS_2_VASc, stroke risk score; CKD, chronic kidney disease; EHRA, European Heart Rhythm Association; HAS-BLED, bleeding risk score; SD, standard deviation.

a Reference group.

At baseline, OAC was used by 1328 (84.2%) patients, with 150 (9.5%) receiving dual antithrombotic therapy. OAC use was similar between the systolic BP groups (Table 2). Most patients (74.9%) received nonvitamin K antagonist oral anticoagulants (NOAC), and 9.3% received a vitamin K antagonist (VKA), with no significant differences between the study groups. At 1-year follow up, antithrombotic therapy use was similar between the systolic BP groups: NOAC 79.2% vs. VKA 8.0%. Beta-blockers were less often used in the group with systolic BP ≥160 mmHg (60.0%) (Table 1, Supplemental Digital Content). The use of digoxin was highest in patients with systolic BP <110 mmHg (22.4%) and 110–119 mmHg (22.5%) – the groups with the highest rate of heart failure. The use of diuretics and aldosterone blockers was more prevalent in those with systolic BP <110 mmHg (42.4% and 11.5%, respectively) and 110–119 mmHg (42.7% and 9.1%, respectively). Those with systolic BP <110 mmHg had the lowest use of calcium channel blockers (7.9%). Use of other medications recorded was similar between systolic BP groups.

**TABLE 2:** Antithrombotic therapy use overall and by baseline systolic blood pressure group

	Study groups according to baseline systolic blood pressure							
Medications, *n* (%)	All groups (*n* = 1580)	120–129 mmHg^a^ (*n* = 289)	<110 mmHg (*n* = 165)	110–119 mmHg (*n* = 254)	130–139 mmHg (*n* = 321)	140–159 mmHg (*n* = 385)	≥160 mmHg (*n* = 166)	*P*-value
Antithrombotic therapy at baseline								
Antiplatelet therapy at baseline (*n* = 1576)								0.30
None	1353 (85.9)	252 (87.8)	140 (84.9)	217 (85.4)	278 (86.9)	329 (85.7)	137 (82.5)	
Aspirin alone	129 (8.2)	26 (9.1)	13 (7.9)	21 (8.3)	25 (7.8)	29 (7.6)	15 (9.0)	
Other monotherapy	52 (3.3)	6 (2.1)	5 (3.0)	9 (3.5)	10 (3.1)	18 (4.7)	4 (2.4)	
Dual antiplatelets	42 (2.7)	3 (1.1)	7 (4.2)	7 (2.8)	7 (2.2)	8 (2.1)	10 (6.0)	
Oral anticoagulant (*n* = 1577):	1328 (84.2)	240 (83.3)	145 (87.9)	224 (88.2)	268 (83.8)	314 (81.8)	137 (82.5)	0.22
VKA	147 (9.3)	28 (9.7)	17 (10.3)	24 (9.5)	30 (9.4)	27 (7.0)	21 (12.7)	0.32
NOAC	1181 (74.9)	212 (73.6)	128 (77.6)	200 (78.7)	238 (74.4)	287 (74.7)	116 (69.9)	
Dual antithrombotic (*n* = 1576)	150 (9.5)	25 (8.7)	22 (13.3)	29 (11.4)	26 (8.1)	30 (7.8)	18 (10.8)	0.28
Antithrombotic therapy at 1 year								
Antiplatelet therapy at 1 year (*n* = 1302)								0.14
None	1214 (93.2)	213 (93.0)	118 (92.2)	189 (91.8)	270 (96.1)	301 (93.8)	123 (89.8)	
Aspirin alone	45 (3.5)	9 (3.9)	6 (4.7)	9 (4.4)	6 (2.1)	7 (2.2)	8 (5.8)	
Other monotherapy	35 (2.7)	7 (3.1)	2 (1.6)	8 (3.9)	5 (1.8)	8 (2.5)	5 (3.7)	
Dual antiplatelets	8 (0.6)	0	2 (1.6)	0	0	5 (1.6)	1 (0.7)	
Oral anticoagulant (*n* = 1302):	1135 (87.2)	195 (85.2)	116 (90.6)	186 (89.9)	243 (86.5)	278 (86.9)	117 (87.2)	0.54
VKA	104 (8.0)	15 (6.6)	9 (7.0)	16 (7.7)	29 (10.3)	21 (6.6)	14 (10.2)	0.52
NOAC	1031 (79.2)	180 (78.6)	107 (83.6)	170 (82.1)	214 (76.2)	257 (80.3)	103 (75.2)	
Dual antithrombotic (*n* = 1576)	60 (4.6)	14 (6.1)	7 (5.5)	16 (7.7)	6 (2.1)	10 (3.1)	7 (5.1)	0.12

NOAC, nonvitamin K antagonist oral anticoagulant; OAC, oral anticoagulant; VKA, vitamin K antagonist.

a Reference group.

The vital status at 1-year follow-up was known for 1524 (96.5%) participants: 75 patients (4.5%) died, with significantly higher (12.2%) mortality observed in patients with systolic BP <110 mmHg (Table 3). There were 19 (1.4%) thromboembolic events, including 7 (0.5%) ischaemic strokes, 8 (0.6%) TIAs and 4 (0.3%) pulmonary embolisms or DVTs. Haemorrhagic events occurred in 20 patients (1.5%), including 6 (0.4%) intracranial and 14 (1.0%) major extracranial bleeding events. No significant differences were observed in the rate of thromboembolic and haemorrhagic events between the systolic BP groups.

**TABLE 3:** Clinical outcomes at 1-year follow-up overall and by baseline systolic blood pressure groups

	Study groups according to baseline systolic blood pressure							
Outcomes, *n* (%)	All groups (*n* = 1524)^a^	120–129 mmHg^b^ (*n* = 278)	<110 mmHg (*n* = 156)	110–119 mmHg (*n* = 245)	130–139 mmHg (*n* = 313)	140–159 mmHg (*n* = 372)	≥160 mmHg (*n* = 160)	*P*-value
All-cause mortality	75 (4.9)	15 (5.4)	19 (12.2)	10 (4.1)	8 (2.6)	15 (4.0)	8 (5.0)	**<0.001**
Thromboembolic events (*n* = 1371)	19 (1.4)	2 (0.8)	2 (1.4)	1 (0.5)	9 (3.1)	3 (0.9)	2 (1.4)	0.22
Ischaemic stroke	7 (0.5)	1 (0.4)	1 (0.7)	0	4 (1.4)	0	1 (0.7)	0.19
Transient ischaemic attack	8 (0.6)	0	1 (0.7)	1 (0.5)	3 (1.0)	3 (0.9)	0	0.56
Pulmonary embolism/DVT	4 (0.3)	1 (0.4)	0	0	2 (0.7)	0	1 (0.7)	0.47
Haemorrhagic events (*n* = 1354)	20 (1.5)	5 (2.1)	3 (2.1)	0	5 (1.7)	6 (1.8)	1 (0.7)	0.40
Intracranial	6 (0.4)	3 (1.2)	2 (1.4)	0	0	1 (0.3)	0	0.09
Major extracranial	14 (1.0)	2 (0.8)	1 (0.7)	0	5 (1.7)	5 (1.5)	1 (0.7)	0.44

DVT, deep vein thrombosis.

a This includes 138 patients for whom only vital status (all alive) was available.

b Reference group.

**FIGURE 1:**
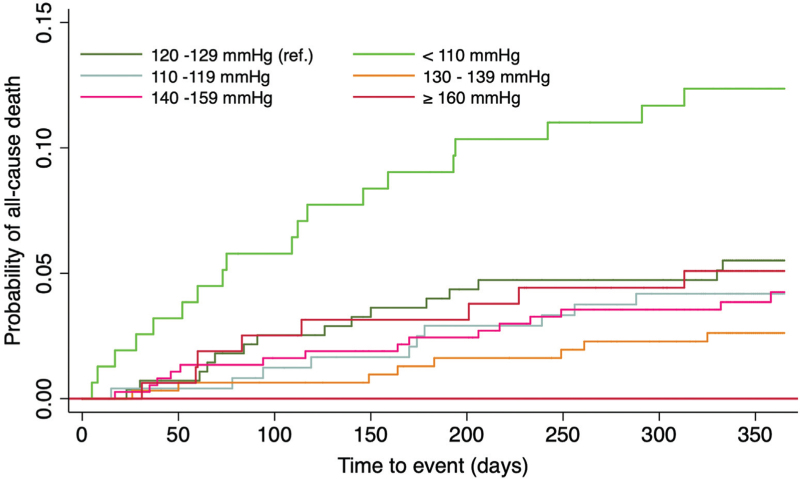
Kaplan–Meier curves for all-cause death by systolic blood pressure groups.

Kaplan–Meier curves showed significantly lower survival in the group with systolic BP <110 mmHg (Fig. 1), with no statistically significant differences between other BP groups (Fig. 1). In univariate Cox regression, predictors of all-cause death were systolic BP<110 mmHg (HR 2.36; 95% CI 1.20–4.64), eGFR (HR 0.99; 95% CI 0.98–0.996), CHA_2_DS_2_-VASc score (HR 1.36; 95% CI 1.20–1.55), and age (HR 1.05; 95% CI 1.03–1.08) (Table 4). Adjusted Cox regression showed that independent predictors of all-cause death were systolic BP <110 mmHg (aHR 0.71; 95% CI 1.73–54.5) and higher CHA_2_DS_2_-VASc score (aHR 1.33; 95% CI 1.12–1.56) (Table 4). In addition, OAC use was associated with a reduced risk of all-cause death (aHR 0.51; 95% CI 0.29–0.92). There was no interaction between BP groups and eGFR. In adjusted Cox regression, independent predictors of thromboembolic events were higher CHA_2_DS_2_-VASc score (aHR 1.53; 95% CI 1.10–2.12, *P* = 0.01) and lack of OAC use (aHR 0.31; 95% CI 0.11–0.92, *P* = 0.04). There was no statistically significant interaction between the systolic BP groups and eGFR for prediction of thromboembolic events. No independent predictors of bleeding events were found (data not shown).

**TABLE 4:** Predictors of all-cause death at 12-month among the AF-GEN-UK cohort

Predictors	Unadjusted hazard ratio (95% CI)	*P*-value	Adjusted hazard ratio (95% CI)	*P*-value
All-cause death				
SBP 120/129 mmHg	Reference group		Reference group^a^	
SBP <110 mmHg	2.36 (1.20–4.64)	**0.01**	9.71 (1.73–54.5)	**0.01**
SBP 110–119 mmHg	0.75 (0.34–1.67)	0.48	2.22 (0.30–16.30)	0.43
SBP 130–139 mmHg	0.46 (0.20–1.10)	0.08	0.16 (0.02–1.22)	0.08
SBP 140–159 mmHg	0.74 (0.36–1.52)	0.42	0.64 (0.11–3.56)	0.61
SBP ≥160 mmHg	0.93 (0.39–2.19)	0.86	0.52 (0.05–5.17)	0.58
eGFR	0.99 (0.98–0.996)	**0.004**	1.00 (0.98–1.01)	0.82
CHA_2_DS_2_VASc score	1.36 (1.20–1.55)	**<0.001**	1.33 (1.12–1.56)	**0.001**
OAC use at baseline	0.68 (0.39–1.18)	0.17	0.51 (0.29–0.92)	**0.02**
Age	1.05 (1.03–1.08)	**<0.001**	1.01 (0.97–1.04)	0.71

BP, blood pressure; CHA_2_DS_2_VASc, stroke risk score; eGFR, estimated glomerular filtration rate; OAC, oral anticoagulation; SBP, systolic blood pressure. Model adjusted for age, use of OAC, CHA_2_DS_2_-VASc score, eGFR, systolic BP groups and their interaction with eGFR.

a Interaction: eGFR # systolic BP groups (*P* > 0.05 for all groups).

On univariate Cox regression, diastolic BP<60 mmHg (HR 3.70; 95% CI 1.88–7.29) was a significant predictor of all-cause death (Table 2, Supplemental Digital Content). On adjusted Cox regression, using model 2, independent predictors of all-cause death were CHA_2_DS_2_-VASc score (aHR 1.19; 95% CI 1.01–1.40) and OAC use (aHR 0.52; 95% CI 0.29–0.94). There was no interaction between the diastolic BP categories and eGFR (Table 2, Supplemental Digital Content). In the adjusted Cox regression, using model 2, the only independent predictor of thromboembolic events was a higher CHA_2_DS_2_-VASc score (aHR 1.47; 95% CI 1.06–2.03, *P* = 0.02). The analysis identified no independent predictors of bleeding events in model 2 (data not shown). Diastolic BP categories were not independently predictive of the study outcomes.

## DISCUSSION

The analyses demonstrate that in a contemporary UK population, in largely anticoagulated patients with AF, systolic BP <110 mmHg independently predicts all-cause mortality irrespective of baseline kidney function. Based on a multivariate model, there was no statistically significant associations between systolic BP groups and risk of haemorrhagic or thromboembolic events after adjustment for other clinical and demographic characteristics and use of OAC. OAC therapy was strongly associated with a reduction in all-cause death and stroke and likely mitigated the risks associated with higher systolic BP levels.

The ‘paradox’ of higher mortality in people with lower systolic BP likely reflects poor overall health. The lack of effect of systolic BP categories for cerebrovascular and thromboembolic events supports this but shows that other health issues were likely driving the higher mortality among people with low systolic BP. Nonetheless, the study demonstrates associations but cannot establish direct causation between lower BP and mortality, as opposed to the effects of multimorbidity, polypharmacy and frailty on outcomes [16–18]. This is important given the clinical complexity associated with AF patients, with implications for treatments and outcomes [18–20]. This is evident by the higher proportion of patients with heart failure and the corresponding higher use of digoxin and diuretics in the group with the lowest systolic BP. While establishing causality would require a treatment intervention and is beyond the scope of this study, the findings suggest that avoiding excessive BP reduction in adequately anticoagulated AF, irrespective of renal function should be advocated. The lack of significant interaction between systolic BP and renal function for prognostication in AF may reflect the bidirectional effects of BP in this population. On one hand, lower systolic BP may diminish renal perfusion, leading to a drop in glomerular filtration. Conversely, lower BP can have a renal-protective effect, but these benefits could not overcome the net risks of excessive BP reduction for overall survival. Effects of BP-lowering treatments in people with worsening renal function may interact with diastolic (or often systolic) cardiac function compromised by AF. However, it is unlikely that BP-lowering drugs specifically influenced the results of this study, and there was no excess in prescribing of these drugs in the group with systolic BP less than 110 mmHg.

Another possible negative impact of excessive BP reduction on all-cause death is the risk associated with very low diastolic BP <60 mmHg [21], which is linked to the reduction in myocardial perfusion, especially in patients with coronary artery disease. In our population, diastolic BP was not an independent predictor of outcomes. This may be because the prevalence coronary artery disease was similar between the study groups.

Elevated BP is a modifiable risk factor for bleeding when on OAC therapy, and it is part of the current bleeding scoring scores. However, a recent posthoc analysis of the RE-LY trial showed that AF patients anticoagulated with VKA or NOAC had an increased mortality risk if systolic BP was <120 mmHg and risk was increased further with a systolic BP <110 mmHg [22]. That was true for baseline and mean-achieved BP values, with the latter being a stronger predictor of higher risk. The latter was also true for the systolic BP>160mmHg in the RE-LY trial [22], but was not observed in our study. The analysis of the RE-LY population accounted for kidney function but not the potential interaction with BP values. In our data, only the group with systolic BP<110mmHg had a higher risk of all-cause mortality.

The Korean National Health Insurance Service data showed that in OAC naïve patients with AF, those with systolic BP 120–129 mmHg were at the lowest risk of death, ischaemic stroke and cardiovascular outcomes [9]. One study in patients with a broader spectrum of kidney function and hypertension but no AF showed that those with systolic BP less <120 mmHg had a higher mortality risk in comparison to those with systolic BP of 120–139 mmHg [23]. The advantage of our analysis is the real-world cohort of patients with AF treated within the same healthcare system, following the same recommendations and offers valuable practical insight.

A recent European consensus document has focused on advances in hypertension management in patients with cardiac arrhythmias [24]. There is a consensus that strict BP control (systolic BP <140 mmHg) can reduce the risk of AF, however this goal is not always easily achieved in clinical practice, and the document does not specify the lowest desirable BP limit. A recent analysis of patients with AF and hypertension, mostly older men (mean age 76 years, 54% men) showed that those who had frequent visits to the same GP achieved better BP control [25]. This study highlights the importance of continuity of care as part of the current holistic approach to AF management [26,27], as recommended in guidelines [28]. Our study further indicates that ongoing monitoring of BP is needed to avoid low systolic BP in people with AF.

### Limitations

The AF-GEN UK registry is a large contemporary cohort but predominantly includes patients managed in secondary care, mainly by cardiologists and may be less reflective of patient management in general practice. However, primary and secondary care in the UK follow the same clinical recommendations and treatment initiated by cardiologists is followed in general practice, making the findings more generalizable. In the UK, the registry was extended to allow recruitment from general practice and other noncardiology specialties, further reducing the ‘speciality’ bias, and the registry provides a snapshot of current practice at the time of the data collection (June 2017–June 2018) and follow-up (June 2018–June 2019). Only 169 (11%) patients had repeat BP values recorded during follow up, which was insufficient for analyses of BP trajectories and to sufficiently assess how patient BP management would impact the study outcomes. The study did not include auditing of BP measurement. Most participants were of white ethnic background and therefore generalization of the findings to other ethnic groups should be made with caution. The study included few patients with advanced CKD, and therefore conclusions about optimal BP targets in these patients are beyond the scope of the analysis. Finally, it was beyond the scope of the registry to record a broader spectrum of co-morbidities to account for them in the study modelling.

## CONCLUSIONS

In largely anticoagulated cohort of people with AF, SBP <110 mmHg is independently predictive of all-cause death, with no significant interaction between SBP and renal (dys)function. This may reflect general poor health and/or excessive antihypertensive therapy, which should be avoided.

## ACKNOWLEDGEMENTS

We are grateful to all the study participants and sites for their time and commitment for the registry. The list of sites and principal investigators are provided in Supplementary material online.

Part of the work was presented at the European Society of Cardiology Congress 2022 and at the BIHS annual scientific meeting 2022.

### Conflicts of interest

The project was supported by the BMS/Pfizer European Thrombosis Investigator Initiated Research Program. The funder had no direct involvement in this study. A.S. has no conflicts of interest to declare. G.Y.H.L.: Consultant and speaker for BMS/Pfizer, Boehringer Ingelheim and Daiichi-Sankyo. No fees are received personally. D.A.L. has received investigator-initiated educational grants from Bristol-Myers Squibb (BMS) and Pfizer, has been a speaker for Bayer, Boehringer Ingelheim, and BMS/Pfizer and has consulted for BMS, and Boehringer Ingelheim.

## Supplementary Material

Supplemental Digital Content
