# Supplementary material for: Relationship between systolic blood pressure and renal function on clinical outcomes in patients with atrial fibrillation: a report from the prospective AF-GEN-UK Registry
Source: J Hypertens. 2024 Sep 23;42(12):2148–54. doi: 10.1097/HJH.0000000000003856 (PMC11556885; doi:10.1097/HJH.0000000000003856)
Supplement: Supplemental Digital Content [file jhype-42-2148-s001.docx]

**Supplementary Table 1.** Medications used at time of enrolment overall and by systolic blood pressure group

| **Medications, n (%)** | **Study groups according to baseline systolic blood pressure** | | | | | | | |
| --- | --- | --- | --- | --- | --- | --- | --- | --- |
|  | All groups  (n = 1580) | 120-129 mmHg*  (n = 289) | <110  mmHg  (n = 165) | 110-119 mmHg  (n = 254) | 130-139 mmHg  (n = 321) | 140-159 mmHg  (n = 385) | ≥160 mmHg  (n=166) | p-value |
| ACE inhibitors (n=1574) | 569 (36.2) | 105 (36.6) | 66 (40.2) | 78 (30.8) | 112 (34.9) | 141 (36.7) | 67 (40.6) | 0.31 |
| ARBs (n=1574) | 211 (13.4) | 35 (12.2) | 18 (10.9) | 35 (13.9) | 40 (12.5) | 48 (12.5) | 35 (21.2) | 0.06 |
| Beta blockers (n=1574) | 1087 (69.1) | 206 (71.8) | 122 (73.9) | 196 (77.5) | 218 (67.9) | 246 (64.2) | 99 (60.0) | **0.001** |
| Digoxin (n=1575) | 232 (14.7) | 38 (13.2) | 37 (22.4) | 57 (22.5) | 33 (10.3) | 54(14.1) | 13 (7.9) | **<0.001** |
| Diuretics (n=1575) | 509 (32.3) | 93 (32.4) | 70 (42.4) | 108 (42.7) | 91 (28.4) | 105 (27.3) | 42 (25.5) | **<0.001** |
| Aldosterone blockers (n=1575) | 103 (6.5) | 18 (6.3) | 19 (11.5) | 23 (9.1) | 17 (5.3) | 16 (4.2) | 10 (6.1) | **0.02** |
| CCB (n=1574) | 291 (18.5) | 46 (16.0) | 13 (7.9) | 37 (14.7) | 70 (21.8) | 80 (20.8) | 45 (27.3) | **<0.001** |
| Statins (n=1573) | 743 (47.2) | 125 (43.6) | 79 (48.2) | 117 (46.3) | 153 (47.7) | 190 (49.6) | 79 (47.9) | 0.76 |
| Oral antidiabetics (n=1575) | 211 (13.4) | 48 (16.7) | 25 (15.2) | 33 (13.0) | 43 (13.4) | 43(11.2) | 19 (11.5) | 0.38 |
| Insulin (n=1575) | 54 (3.4) | 15 (5.2) | 5 (3.0) | 8 (3.2) | 10 (3.1) | 12 (3.1) | 4 (2.4) | 0.60 |
| Thyroid hormones (n=1575) | 120 (7.6) | 19 (6.6) | 16 (9.7) | 22 (8.7) | 21 (6.5) | 28 (7.3) | 14 (8.5) | 0.76 |
| Thyroid-suppressing (n=1575) | 13 (0.8) | 1 (0.4) | 2 (1.2) | 2 (0.8) | 2 (0.6) | 6 (1.6) | 0 | 0.39 |
| NSAIDs (n=1574) | 37 (2.4) | 6 (2.1) | 4 (2.4) | 4(1.6) | 12 (3.7) | 6 (1.6) | 5 (3.0) | 0.44 |

ACE, angiotensin converting enzyme inhibitors; ARB, aldosterone receptor blockers; CCB, Calcium channel blockers; NSAIDs, non-steroidal anti-inflammatory drugs; *reference group.

**Supplementary Table 2.** Association of diastolic blood pressure with all-cause death at 12-month among the AF-GEN-UK cohort

| **Predictors** | **Unadjusted**  **Hazard Ratio (95% CI)** | **p-value** | **Adjusted**  **Hazard Ratio**  **(95% CI)** | **p-value** |
| --- | --- | --- | --- | --- |
| **All-cause death** | | | | |
| DBP 70/79 mmHg | reference | | reference * | |
| DBP <60 mmHg | 3.70 (1.88-7.29) | **<0.001** | 3.50 (0.62-19.7) | 0.16 |
| DBP 60-69 mmHg | 1.14 (0.56-2.32) | 0.71 | 6.36 (0.82-49.4) | 0.08 |
| DBP 80-89 mmHg | 0.85 (0.44-1.65) | 0.63 | 0.60 (0.11-3.21) | 0.55 |
| DBP ≥90 mmHg | 0.82 (0.40-1.68) | 0.58 | 0.40 (0.07-2.26) | 0.30 |
| eGFR | 0.99 (0.98-0.996) | **0.004** | 0.99 (0.98-1.01) | 0.38 |
| CHA_2_DS_2_VASc score | 1.36 (1.20-1.55) | **<0.001** | 1.19 (1.01-1.40) | **0.04** |
| OAC use at baseline | 0.68 (0.39-1.18) | 0.17 | 0.52 (0.29-0.94) | **0.03** |
| Age | 1.05 (1.03-1.08) | **<0.001** | 1.01 (0.98-1.04) | 0.58 |

CHA_2_DS_2_VASc, stroke risk score; eGFR, estimated glomerular filtration rate; OAC, oral anticoagulation; DBP, diastolic blood pressure.

Model adjusted for age, use of OAC, CHA_2_DS_2_-VASc score, eGFR, diastolic blood pressure categories and their interaction with eGFR, *interaction: eGFR # diastolic blood pressure categories (p>0.05 for all groups).

**List of sites and Principal Investigators**

**GP practices:**

Alconbury and Brampton Surgeries [Dr Duncan Outram]; Armley Primary Care Federation [Dr Gary Lees]; Ashcroft Surgery [Dr Craig Frame]; Ashfields Primary Care Centre [Dr Neil Paul]; Ash Trees Surgery [Dr Harinder Mehta]; Bay Medical Group [Dr Muhammad Akhtar]; Bodey Medical Centre [Dr Simon Rogers]; Bridge Road Surgery [Dr Martin Aylward]; Broadshires Health Centre [Dr Christine A’Court]; Brookhouse Surgery and Shifa Surgery [Dr Tanveer Ahmed]; Buckden & Little Paxton Surgery [Dr Chris Newark]; Carlisle Healthcare CCG North Cumbria [Dr Stacey Fisher]; Claughton Medical Centre [Dr Peter Arthur]; Clifton Medical Centre [Dr Matthew Capehorn]; Colchester Medical Practice [Dr Costas Paschalides]; Colte Partnership [Dr Ayo Ajala]; Cromer Group Practice [Dr Raife Oliver]; East Coast Community Healthcare [Dr Andrew Emerson]; Eden Court Medical Centre [Dr Richard Edwards]; Eve Hill Medical Practice [Dr David Shukla]; Eynsham Medical Group [Dr Ian Binnian]; Firdale Medical Centre [Dr Susan Brown]; Garswood Surgery [Dr David Lawson]; Gosforth Hill Medical Centre [Dr Matthew Wallard]; Hall Green Health [Dr Nashat Qamar]; Kiltearn Medical Centre [Dr Carolyn Paul]; Lancaster Medical Practice [Dr Andrew Gallagher]; Magdalen Medical Practice [Dr Kate Milne]; Meadowbrook Surgery [Dr Iqbal More]; Minden Family Practices [Dr Karishma Singh]; Modality Partnership- Wokingham Medical Practice [Dr Zisham Ali]; Mosslands Medical Practice [Dr Neil Bates]; MyHealth [Dr Fran Adams]; Northenden Group Practice [Dr Naresh Kanumilli]; Pendle View [Dr Umesh Chauhan]; Peel House Medical Practice [no PI listed]; Pickering Medical Practice [Dr Swaminathan Thiagarajan]; Pocklington Group Practice [Dr James Laing]; Priory Gardens [Dr Nasir Hanna]; Riverside Surgery [Dr Avinash Pillai]; Rosedale Surgery [Dr Maarten Derks]; Sides Medical Centre [Dr Laurence Cribbin]; South Chadderton Health Centre [Dr Anita Sharma]; Springbank Surgery [Dr Richard Tatham]; Staithe Surgery [Dr Satish Singh]; St Gabriel's Medical Centre [Dr Richard Deacon]; Staploe Medical Centre [Dr Anthony Gunstone]; The Arch Medical Practice [Dr Steven Colabella]; The Gill Medical Centre [Dr Nicholas Browne]; The Leeds Road Practice [Dr Peter Banks]; The Peninsula Practice [Dr Lindsay Crockett]; The Riverside Practice [Dr Peter Spofforth]; The Village Practice [Dr Preeti Pandya]; Waterloo Medical Centre [Dr John Calvert]; West Timperley Medical Centre [Dr Catherine Effingham]; Winterton Medical Practice [Dr Rebecca Clark]; Woodlands Medical Practice [Dr Susan Kenyon]; Woolpit Health Centre [Dr Richard West]

**NHS Trusts:** Ayrshire and Arran University Hospital [Dr James McGowan]; Ashford & St Peters Hospitals NHS Trust [Dr Mark Williams]; Belfast Health and Social Care Trust [Dr Andrew Moriatry]; Betsi Cadwaladr University Health Board [Dr Satheesh Balakrishnan Nair]; Blackpool Teaching Hospitals NHS Foundation Trust [Dr Gavin Galasko]; Calderdale & Huddersfield NHS Foundation Trust [Dr Simon Grant]; County Durham & Darlington NHS Foundation Trust [Dr Annie Abraham]; East Lancashire Hospitals NHS Trust (Royal Blackburn) [Dr Scot Garg]; East Sussex Healthcare Trust (Conquest Hospital) [Dr Christina Elorz]; Guys & St Thomas NHS Trust [Dr Laura Hunter]; Hereford County Hospital [Dr Colin Jenkins]; Lancashire Teaching Foundation Trust (Royal Preston) [Dr Tahir Nazir]; Manchester University NHS Foundation Trust [Dr Fozia Ahmed]; North Cumbria University Hospitals NHS Trust [Dr Louise Buchanan]; Northern Devon Healthcare NHS Trust [Dr Svetlana Kaminskiene]; Northumbria Healthcare NHS Trust [Dr Honey Thomas]; Pennine Acute Hospitals NHS Trust [Dr Jolanta Sobolewska]; Plymouth Hospitals NHS Trust [Dr A Mohd Nor]; Poole Hospitals NHS Trust [Dr Christopher Boos]; Royal Berkshire NHS Foundation Trust [Dr Varun Nelatur]; Royal Cornwall Hospitals NHS Trust [Dr Frances Harrington]; Royal Devon & Exeter Healthcare NHS Trust [Dr Andrew Sharp]; Salford Royal Hospital NHS Foundation Trust [Dr Alan Fitchet]; Salisbury NHS Foundation Trust [Dr Manas Sinha]; Sandwell & West Birmingham Hospitals NHS Trust [Prof Gregory Lip]; Shrewsbury & Telford Hospital NHS Trust [Dr Thomas Ingram]; Southampton University Hospital Trust [Dr Paul Roberts]; South Tees Hospitals NHS Foundation Trust [Dr Samer Al Hussayni]; South Tyneside NHS Foundation Trust [Dr Abdul Nasser]; Southend University Hospital NHS Foundation Trust [Dr Paul Guyler]; Sunderland NHS Foundation Trust [Dr Min Myint]; Surrey & Sussex Healthcare NHS Trust [Dr Nadkumar Gandhi]; Tameside and Glossop Integrated Care NHS Foundation Trust [Dr Edward Jude]; Taunton And Somerset NHS Foundation [Dr David Beacock]; Torbay & South Devon NHS Foundation Trust [Dr Dirk Felmeden]; University Hospitals South Manchester NHS Foundation Trust [Dr Ed Gamble]; University Hospitals North Midlands NHS Trust [Dr Indira Natarajan]; University Hospital North Tees [Dr Ignaio Cardona]; Warrington and Halton Hospitals NHS Foundation Trust [Dr Hrvojka Marija Zeljko]; West Hertfordshire Hospital NHS Trust [Dr Masood Khan]; Wrightington, Wigan & Leigh NHS Trust [Dr Habib Rehman]; Yeovil District Hospitals NHS Foundation Trust [Dr Andrew Broadley]; York Teaching Hospital NHS Trust [Dr Rasheed Hossain]
